# Supplementary material for: Timely initiation of breastfeeding and associated factors among mothers having children less than two years of age in sub-Saharan Africa: A multilevel analysis using recent Demographic and Health Surveys data
Source: PLoS One. 2021 Mar 23;16(3):e0248976. doi: 10.1371/journal.pone.0248976 (PMC7987153; doi:10.1371/journal.pone.0248976)
Supplement: S1 Table — (DOCX) [file pone.0248976.s001.docx]

**S1 Table. The 35 SSA countries used for analysis and their sample size.**

| **Country** | **Year of study** | **Weighted frequency** | **percentage** |
| --- | --- | --- | --- |
| Angola | 2015/16 | 2593 | 2.55 |
| Burkina Faso | 2010 | 5739 | 5.64 |
| Benin | 2017/18 | 10309 | 10.13 |
| Burundi | 2016/17 | 5159 | 5.07 |
| DR Congo | 2013/14 | 6938 | 6.81 |
| Congo | 2011/12 | 3447 | 3.39 |
| Cote D’Ivoire | 2011/12 | 2923 | 2.87 |
| Cameroon | 2018/19 | 3697 | 3.63 |
| Ethiopia | 2016 | 4050 | 3.98 |
| Gabon | 2012 | 1262 | 1.24 |
| Ghana | 2014 | 1153 | 1.13 |
| Gambia | 2013 | 1585 | 1.56 |
| Guinea | 2018 | 1358 | 1.33 |
| Kenya | 2014 | 3383 | 3.32 |
| Comoros | 2012 | 1177 | 1.16 |
| Liberia | 2013 | 1266 | 1.24 |
| Lesotho | 2014 | 619 | 0.61 |
| Madagascar | 2008 | 2378 | 2.34 |
| Mali | 2018 | 3850 | 3.78 |
| Malawi | 2015/16 | 2131 | 2.09 |
| Mozambique | 2011 | 4550 | 4.47 |
| Nigeria | 2018 | 4801 | 4.72 |
| Niger | 2012 | 2351 | 2.31 |
| Namibia | 2013 | 818 | 0.8 |
| Rwanda | 2014/15 | 1510 | 1.48 |
| Sierra Leone | 2013 | 2117 | 2.08 |
| Senegal | 2018 | 2340 | 2.3 |
| Sao Tome Principe | 2008/09 | 700 | 0.69 |
| Chad | 2014/15 | 4090 | 4.02 |
| Togo | 2013/14 | 1312 | 1.29 |
| Tanzania | 2015/16 | 3956 | 3.89 |
| Uganda | 2016 | 1807 | 1.77 |
| South Africa | 2016 | 499 | 0.49 |
| Zambia | 2018 | 3691 | 3.63 |
| Zimbabwe | 2015 | 2258 | 2.22 |
| Total | 101,815 | | 100 |
