# Supplementary material for: Timely initiation of breastfeeding and associated factors among mothers having children less than two years of age in sub-Saharan Africa: A multilevel analysis using recent Demographic and Health Surveys data
Source: PLoS One. 2021 Mar 23;16(3):e0248976. doi: 10.1371/journal.pone.0248976 (PMC7987153; doi:10.1371/journal.pone.0248976)
Supplement: Attachment — Submitted filename: RESPONSE TO REVIEWER.docx [file pone.0248976.s002.docx]

**Date: December 2020**

**Point by point response to editor and reviewers comment**

**Title:** Timely initiation breastfeeding and its associated factors among mothers with under 24 months living children in Sub Saharan Africa: a multilevel analysis using DHS Data from 35 Sub Saharan African countries

**Manuscript number:** PONE-D-20-27660

**Dear editor and Reviewers:** We really thank you for your valuable comments for the betterment of our manuscript. Your concerns and questions as well as suggestions are addressed in the revised manuscript (see the point-by-point response).

**Response to editor comment**

1. Please ensure that your manuscript meets PLOS ONE's style requirements, including those for file naming.

**Author’s response**: Thank you. The revised manuscript is prepared according to PLOS ONE's style.

2. We suggest you thoroughly copyedit your manuscript for language usage, spelling, and grammar.

**Author’s response**: The revised manuscript is extensively edited for language usage, spelling, and grammar after consulting our colleagues who had MA degree in “teaching English as foreign language” and who had many years experience in the area of literature at University of Gondar. A copy of our manuscript showing the changes is indicated by using track changes (See supporting information file).

**Response to reviewers comment**

**Reviewer #1:**

1. **Title:**
   The title could be more succinct. Note that the location (Sub Saharan Africa) is repeated.

**Author’s response:** Thank you for the important comment you raised. We amended the title accordingly in the revised manuscript.

2. **Abstract:**
   -Please, note that the abstract exceeds the number of words allowed.

**Author’s response:** Thank you. According to PLOS ONE journal submission guideline, the number of words in the abstract should not be above 500 and we prepared based on this and the total words in the abstract is below 500 (around 360) in the revised manuscript.

-Kindly check if all the factors you described in the results section are community level factors. See that you presented “Among community level factors” and “of community level factors”.

**Author’s response:** Thank you. It was to mean “Among individual level factors” and we amended it in the revised manuscript.

-The conclusion paragraphs repeat the information presented in results.

**Author’s response:** Thank you. We consider your comment in the revised manuscript**.**

3. **Background:**
   -Please, consider if in addition to the ranges it is possible to inform the prevalence of TIBF in SSA.

**Author’s response:** Thank you. We incorporated the prevalence of TIBF in SSA in the revised manuscript (see line 61 & 62).

-Does “Residence” mean area of residence?

**Author’s response:** Yes, it was to mean “place of residence” or “area of residence” and we amend residence to place of residence in the revised manuscript.

-When you explain that previous studies only considered a single country, are you talking about studies performed in Africa? Please review this paragraph. I do not think that the limitation of the previous studies has a connection with the previous sentence.

**Author’s response:** Thank you. We amended these statements in the revised manuscript (see line 67-72).

4. **Methods:**
   -What exactly does “KR” mean? Kids, what else?

**Author’s response:** Thank you. It was to mean Kids data set and in the revised manuscript, we remove the abbreviation since we hope it add nothing.

-In the dependent variable paragraph, please, add the definition of TIBF and how it was measured. This information was mentioned in the discussion section (maternal report).

**Author’s response:** Thank you. We add the definition of TIBF in the revised manuscript.

-In the independent variables paragraph, could you please add the categorization used for each variable in the analyses?

**Author’s response:** Thank you. We incorporate the categorization of each variables (see table 1 in the revised manuscript).

-Why did you categorize maternal age in that way? Please, explain the rationale for those ranges. Consider recategorizing the variable into less categories since in the discussion section you describe just "older and younger mothers".

**Author’s response:** Dear reviewer thank you for raising this important issue. We did not re-categorize the age group for our analysis; we used the DHS categorization as it is. Many studies done on DHS data use this categorization. In addition, in the interpretation and in the discussion section we consider your comment. Dear reviewer we say older age because we compared with younger age groups (all consecutive age groups are older as compared with 15-19 age groups). If this does not convince you, we are open to consider your concern again and categorize to less categories.

-Please, note that in the independent variables paragraph you said “four community level variables” but there were 5 mentioned (including residence).

**Author’s response:** Thank you. We amend it to read “Six community level variables” in the revised manuscript.

-Regarding the community level factors, based on what did you set a 50% cut-off point?

**Author’s response:** Since the distribution of the community level variables were not normally distributed (to use the mean value), we used the national median value (50%) as a cut-off point.

-Please, note that in table 1 you also presented a “Community poverty level” that was not described in methods section.

**Author’s response:** Thank you very much. We consider your comment (we added community poverty level in the revised manuscript, which was missed in the method section).

-In table 3, What does AOR mean? It was not mentioned in statistical analysis section. Besides, is the confidence interval 95%? Please add this information.

**Author’s response:** Thank you. We consider your comment in the data management and statistical analysis section of the revised manuscript.

5. **Results:**
   -The following information was given before: “total weighted sample of 101815 women was used for our analysis.”

**Author’s response:** Thank you for the comment. We avoided the redundancy in the revised manuscript.

-Please check the following sentence “Most (40.46%) of the respondents had no formal education” note that most of the participants (59.5% ) had some formal education.

**Author’s response:** Thank you. We amended it per your recommendation.

-Observe that the main purpose of the study is to investigate associated factor of TIBF. However, in this section you used the following title: “Determinants of timely initiation of breastfeeding…”

**Author’s response:** Thank you for the comment. We amended to read, “Factors associated with timely initiation of breastfeeding …” in the revised manuscript.

-Concerning “…those from a higher risk cluster had 1.37 times higher odds of TIBF as compared to those individuals who come from the lower risk cluster” Please, clarify what a higher risk refers to? In this case higher odds of TIBF would be beneficial/expected, right? It seems contradictory to talk about higher risk of having 1.37 times higher odds of TIBF. See if you can reformulate the sentence.

**Author’s response:** Thank you for the comment. We consider your comment and amend accordingly in the revised manuscript (see line 172-175).

-The information given from line 172 to 177 is repeating what was described from line 178 on.

**Author’s response:** Thank you. We consider your comment and remove redundancy in the revised manuscript.

-In table 3, I think that null model column is not necessary.

**Author’s response:** Dear reviewer thank you for the comment. It is the case in the multilevel model and if we remove the null model in the table, the table will be incomplete and may mislead the readers. Therefore, we prefer to put it in Table 3. We are open to remove the column showing the null model if you are not still convinced.

-Please, review the labels of the categories in the wealth index. You can name the quintiles from the first (lowest) to the fifth (highest), instead of “the lowest and the second quintile”. It will be useful for describing this factor in the results and discussion section.

**Author’s response:** Thank you. We consider your comment in the revised manuscript.

6. **Discussion and conclusion:**
   -Please, conclude the first paragraph of the discussion. You compared the results of the study with other literature, but what does 58% of TIBF mean? Does it comply with the recommendation? (WHO reference: Infant and Young Child Feeding. A tool for assessing national practices, policies and programmes. Geneva. 2003).

**Author’s response:** Thank you. We consider your comment in the revised manuscript

-The text from line 214 to 219 has already been described in the results section.

**Author’s response:** Thank you. We consider your comment and avoid redundancy in the revised manuscript.

-In relation to “This is because educated mothers might have exposure to appropriate breastfeeding practices” could you explain it better?

**Author’s response:** Thank you. We explained it in a better way in the revised manuscript.

In relation to “Contrary to our expectation, mothers who have been exposed to media had lower odds of TIBF as compared to their counterparts.” Why “contrary to our expectation”? What about the aggressive advertising of infant formula industry, milk substitutes, teats, and bottles regarding the media?

**Author’s response:** Really thank you for your concern and giving direction. We amended this statement accordingly in the revised manuscript.

-The discussion on community levels of ANC utilization and health facility delivery is not clear. Can you give more details and expand the discussion?

**Author’s response:** Thank you. We discussed these community level variables in detail in the revised manuscript.

-Line 292, determinants of TIBF, or associated factors?

**Author’s response:** We amended it to read, “Factors associated with TIBF”

-It is important that the discussion includes not only the comparison of the results with other studies and the authors conclusions, but also the theoretical basis for these conclusions. Please, support all your conclusions with bibliographic references.

**Author’s response:** Thank you. We consider your comment and put references in the revised manuscript accordingly.

-Please, review the conclusion: what was found in the study and the recommended "special emphasis", repeats the information.

**Author’s response:** Thank you again for raising this issue. We revised the conclusion section in the revised manuscript.

7. **References and general comments**
   -Please, check all the references and make sure the institutions names are consistently cited. (Organization WH., WHO., World Health Organization, Unicef., UNICEF.?)

**Author’s response:** We revise the reference section in the revised manuscript.

-Please, remove the space between lines (for example, between line 56 and 57). Check all the text and make sure the format is consistent.

**Author’s response:** Thank you. We check the overall manuscript and we amended any errors encountered.

-Please, avoid the following text structure (mistimed/unintended, inappropriate/suboptimal, physicians/midwives). If possible, choose one word or use “and” when necessary.

**Author’s response:** Thank you. We consider your comment in the revised manuscript.

-Overall: I recommend editing the text to achieve a more appropriate grammar structure and scientific language.

**Author’s response:** Thank you. We extensively read our manuscript and edit it for language usage, spelling, and grammar after consulting our colleagues and language experts in our university.

**Reviewer #2:**

1. **Abstract**

   Line 19 …’despite its major implication…’ perhaps it should read ‘despite THE major implications of…’ – However, I would suggest having this first line revised in its totality.

**Author’s response:** Thank you. We consider your comment in the revised manuscript.

2. **Methodology**

   The authors have stated that they selected DHS data from 35 countries. Malawi, for example, has the most recent DHS data in 2015-16, why did the authors choose to analyse the 2011 (which I suppose should be 2010 data) instead of the most recent data?

**Author’s response:** Dear reviewer thank you for raising this important concern. We were used the 2015/16 survey data for Malawi, however we reported as we used the 2011 survey data. Therefore, in the revised manuscript and figure, we checked and modify to read, “2015/16”.

The analysis is a pooled one, with 35 different countries having their respective weighting variables according to their sample selection. It would be appropriate for the authors to describe whether they generated pooled weights and briefly explain how this was done.

**Author’s response:** Dear thank you for raising this important issue. We appended the data sets and we apply weighting. That is weighting was conducted using the primary sampling unit variable, stratification variable, and the weight variable after appending the DHS data sets.

Did the authors check for multicollinearity of their models?

**Author’s response:** Thank you very much. Variance inflation factor (VIF) was used to assess Multicollinearity and there was no Multicollinearity between explanatory variables, with a mean VIF of 1.81 (the minimum and the maximum VIF was 1.01 and 3.96 respectively) (see line 113-115).

Under the data analysis section, authors aught to mention that adjusted odds ratio and 95% CI were reported…

**Author’s response:** Thank you. We mentioned it in data management and analysis section of the revised manuscript.

3. **Results**
   This is just a 'cosmetic' comment in the presentation of the results: It would look great if the fixed effects are presented first then random effects later thus Table 2 could be Table 3 while the current Table 3 may be changed to Table 2.

**Author’s response:** Thank you. We accepted your comment in the revised manuscript.

4. **Discussion**
   Line 285-290 – More explanation needed and if possible, provide some refs. Why would women coming from communities with high ANC utilization more likely to develop a norm of early BF initiation?

**Author’s response:** Thank you. We consider your comment in the revised paper. Moreover, women coming from communities with high ANC utilization are more likely to develop a norm of early BF initiation since there is higher probability of sharing information regarding infant feeding practices among mothers in the community.

5. In the conclusion section, the authors have mention that the prevalence of TIBF was relatively lower, ,, lower compared to what? What are they comparing this prevalence to?

**Author’s response:** Thank you. We said low based on the clinical impact (since it is a major public health problem) and comparison with the other previous studies. However, we consider your comment and amend to read, “The prevalence of TIBF in SSA was good, consistent with the WHO rating of the TIBF practice as good”
