# Supplementary material for: Timely initiation of breastfeeding and associated factors among mothers having children less than two years of age in sub-Saharan Africa: A multilevel analysis using recent Demographic and Health Surveys data
Source: PLoS One. 2021 Mar 23;16(3):e0248976. doi: 10.1371/journal.pone.0248976 (PMC7987153; doi:10.1371/journal.pone.0248976)
Supplement: Attachment — Submitted filename: Response to reviewer ver#2.docx [file pone.0248976.s003.docx]

**Date: January 29, 2021**

**Response to editor and reviewer**

**Title:** Timely initiation of breastfeeding and associated factors among mothers having children less than two years of age in sub-Saharan Africa: a multilevel analysis using recent Demographic and Health Surveys Data

**Manuscript number:** PONE-D-20-27660R1

Dear editor and reviewers thank you for raising the important concerns for the betterment of the manuscript. Here is the point-by-point response from the authors.

**Response to editor**

1. As nicely pointed out by Reviewer #2, it is important to consider performing a three-level analysis to assess between-country heterogeneity, in view of the large interval of TIBF among SSA countries.

**Author’s response:** Thank you for the important comment you raised and your recommendation. We have tried to did a three level analysis to encounter the between country heterogeneity and unfortunately the output is not generated (we have convergence problem). We hope that as far as we account community-level heterogeneity the estimates are not that much different. Dear editor, we have acknowledged this as the limitation in the revised manuscript.

2. While TIBF rate in SSA countries was "good" overall, according to WHO all babies (except in few cases where BF is contraindicated) should benefit from breastfeeding early in life. As indicated by Reviewer #1, such a finding should consider the great variation in SSA [note here that results from the three-level analysis could be quite informative] and also the great room for improvements in breastfeeding, including actions for the protection, promotion and support of this practice. Please revise the manuscript throughout.

**Author’s response:** Thank you. We have considered your concern in the revised manuscript. Considering the variations of TIBF in SSA and the WHO recommendation of all babies should benefit from breastfeeding early in life, we amend it to read, “TIBF in SSA countries was low”.

3. After revising your analysis, please make sure to properly reference the hypothesis drawn and/or underlying mechanisms suggested for the observed associations while discussing your findings.

**Author’s response:** Thank you. We have revised the underlying mechanisms suggested for the observed associations while discussing our findings.

4. An extensive review of the abstract is needed (in up to 300 words), as well as of the structure of the discussion section.

**Author’s response:** Thank you. We have considered your comment in the revised manuscript. We minimized the abstract to 300 words and we review and amend the structure of the discussion section.

**Response to reviewer**

Reviewer #1:

1. Please, check with the journal editor the number of words allowed in the abstract. In "Submission guidelines" - https://journals.plos.org/plosone/s/submission-guidelines#loc-abstract - Parts of submission - abstract, says: Not exceed 300 words.
   **Author’s response:** Thank you. We amend the abstract section (we minimize the number of words to 300).
2. Introduction:
   Please, could you review the following sentence? “Even though there is a study conducted on the prevalence of TIBF in SSA [14], without identifying the hindering factors of TIBF, it is unlikely that TIBF can occur and reduce under-five mortality.” Could you briefly explain this better? I think the sentence is not clear.

**Author’s response:** Thank you. We make it clear in the revised manuscript**.**

3. Discussion:
   -Please, review all the paragraphs and support your conclusions with bibliographic references. See an example: “In this study, EBF was higher among multiparous mothers…. Cultural practices of early introduction of teas, water, and foods along with BM, probably have a greater impact on practices with the first child when mothers are not well supported for exclusive breastfeeding (reference). Thus, mothers with previous experience in the breastfeeding process and possibly older, are usually more mature in what concerns care and feeding of a child when compared to primiparous women.”

**Author’s response:** Thank you. We have reviewed all the paragraphs and we have putted the conclusions we made with bibliographic references.

-I thank the authors for the English revision of the text. However, in the discussion section, I think the text could be more fluid. Please note that all paragraphs discussing the associated factors have the same structure: "This is because ..., This may be because ..., This might be because ..., This might be due to ..., This could be because ... ".

**Author’s response:** Really thank you for the important comment you raised. We consider your issue in the revised manuscript.

-I recommend the authors to further discuss the following factors: type of birth (single/multiple) and place of residence.

**Author’s response:** Thank you. The above stated variables are discussed in the revised manuscript.

-Lastly, TIBF rate was good according to WHO (countries are consider good when at least 50% of the babies initiate BF in the first hour of life), but it is important to note that also according to WHO all babies (except when BF is contraindicated - few cases) should benefit from breastfeeding early in life. So, although the TIBF rate found in the study was good, it could be improved, especially because you found huge variation between countries. I think some words about it will complete your idea when you talk about "to plan appropriate strategies and implement interventions." Please, see if this makes sense to you.

**Author’s response:** Thank you for your comment and direction. We have considered your recommendation and revised the statement accordingly.

**Reviewer #2**:

1. Abstract
   Line21-22, the sentence should be rewritten as it stands, it may mean that the SDG on reduction of <5 mortality solely depends on optimal breastfeeding. In as much as optimal breastfeeding is an important component in achieving this SDG goal, there are other indicators such as vaccination that are also important. Maybe authors can just state that ‘optimal breastfeeding is one of the key components that may help achieve reduction of <5 mortality….’

**Author’s response:** Dear reviewer, thank you for the nice comment you raised. We have considered your comment as well as recommendation and amended the statement accordingly.

In the abstract conclusion (Line 42), authors state that the prevalence of TIBF was GOOD in SSA. ‘Good’ based on what standard?

**Author’s response:** Thank you. It is amended to read “The prevalence of TIBF in SSA was low” and we said low based on the huge variations between countries and according to the WHO recommendation that all babies should benefit from breastfeeding early in life**.**

2. Introduction
   Line 70-72 in the introduction section is not clear.

**Author’s response:** Thank you. We make it clear in the revised manuscript (see line 67 and 68).

3. Methods
   - Please state that the most recent DHS data was selected for analysis from each country specifically for those countries that have more than 1 surveys.

**Author’s response:** Thank you. We have considered it in the revised manuscript (see line 76 & 77).

- Line 81-82; ‘after appending….’ I think this is misplaced. Perhaps include it in the data management section.

**Author’s response:** Thank you. We considered your comment in the revised manuscript (The first sentence of the data management and analysis section, line 99).

- I want clarification whether authors constructed new cluster and stratum variables and whether the weights were redefined considering that they had appended the datasets from several countries.

**Author’s response:** Dear reviewer, thank you for the important concern you raised. As you know, the weighting variables are coded in similar in each DHS we have weighted the data using sample weight, v005 that is helpful to adjust over and under sampling and the SVY set command to account for the complex survey design and generalizability of the findings.

4. Results
   For the pooled estimates, did the authors adjust for survey (i.e., country)? I think the results would be more interesting if a variable called ‘country/survey’ is included.

**Dear reviewer:** We have weighted the data using sample weight, v005 ,that is helpful to adjust over and under sampling and the SVY set command to account for the complex survey design and generalizability of the findings.

Follow up suggestion on the above comment: The authors did a two-level analysis, where individuals, and communities were analysed. As this survey pooled several countries, a three-level analysis would have been more interesting to not only observe community-level heterogeneity, but also assess between country heterogeneity

**Author’s response:** Dear reviewer we have weighted the data to adjust sampling and to account for complex survey design. However, we are unable to conduct a three level analysis since we have convergence difficulty (even we tried to conduct a three level analysis to identify both community-level heterogeneity and country heterogeneity but we are unable to get the output). Dear reviewer, many multilevel studies conducted using pooling of the DHS data also adjust for community level heterogeneity only due to similar issue. Therefore, we acknowledge it in the revised manuscript as limitation of our study (see line 273 &274). Hope this convinces you, however, we are open to accept directions and recommendations if you have.
